# *Sida rhomboidea*. Roxb Leaf Extract Down-Regulates Expression of PPARγ2 and Leptin Genes in High Fat Diet Fed C57BL/6J Mice and Retards *in Vitro* 3T3L1 Pre-Adipocyte Differentiation

**DOI:** 10.3390/ijms12074661

**Published:** 2011-07-19

**Authors:** Menaka C. Thounaojam, Ravirajsinh N. Jadeja, Umed V. Ramani, Ranjitsinh V. Devkar, A. V. Ramachandran

**Affiliations:** 1 Division of Phytothrapeutics and Metabolic Endocrinology, Department of Zoology, The M. S. University of Baroda, Gujarat 390002, India; E-Mails: menakachanu@yahoo.com (M.C.T.); rv_jadeja@yahoo.co.in (R.N.J.); av_rama@rediffmail.com (A.V.R.); 2 Department of Animal Biotechnology, College of Veterinary Science and Animal Husbandry, Anand Agriculture University, Anand, Gujarat 388001, India; E-Mail: umedramani@yahoo.co.in

**Keywords:** *Sida rhomboidea.* Roxb, obesity, 3T3L1 cells, PPARγ2, leptin

## Abstract

*Sida rhomboidea*. Roxb leaf extract (SRLE) is being used by the populace of North-East India to alleviate symptoms of diabetes and obesity. We have previously reported its hypolipidemic and anti-diabetic properties. In this study, we report the effect of SRLE on (i) *in vivo* modulation of genes controlling high fat diet (HFD) induced obesity and (ii) *in vitro* 3T3L1 pre-adipocyte differentiation and leptin release. Supplementation with SRLE significantly prevented HFD induced increment in bodyweight, plasma lipids and leptin, visceral adiposity and adipocyte hypertrophy. Also, SRLE supplementation reduced food intake, down regulated PPARγ2, SREBP1c, FAS and LEP expressions and up-regulated CPT-1 in epididymal adipose tissue compared to obese mice. *In vitro* adipogenesis of 3T3L1 pre-adipocytes was significantly retarded in the presence of SRLE extract. Also decreased triglyceride accumulation, leptin release and glyceraldehyde-3-Phosphate dehydrogenase activity along with higher glycerol release without significant alteration of viability of 3T3L1 pre-adipocytes, was recorded. Our findings suggest that prevention of HFD induced visceral adiposity is primarily by down regulation of PPARγ2 and leptin gene expression coupled with attenuation of food intake in C57BL/6J mice. SRLE induced prevention of pre-adipocytes differentiation, and leptin release further substantiated these findings and scientifically validates the potential application of SRLE as a therapeutic agent against obesity.

## 1. Introduction

Obesity, a fast spreading epidemic, is a major contributor to the global burden of chronic disease and disability. Currently, more than one billion adults worldwide are overweight and at least, 300 million of them are clinically obese [1]. Such individuals are maximally prone to type-2 diabetes, cardiovascular disease and hypertension in the long run [2,3]. Induction of obesity in humans is either ‘genetic’ of ‘lifestyle’ related. The latter is however a complex intermix of sedentary lifestyle and a high calorie diet amounting to nutritional overload [4,5]. Synthetic anti-obesity drugs have often been reported to be very costly with some of them also beset with undesirable side effects, thereby necessitating a need to screen natural/herbal products for treating obesity [6]. In recent times, many traditional herbal preparations have been put through a detailed scrutiny to explore their anti-obesity potential and the underlying mechanism of action [7].

*Sida rhomboidea.* Roxb (SR; Fam, Malvaceae) is a weed found in marshy places across India. In Ayurveda, it is known as “*Mahabala*” and is used for treating fever, heart diseases, burning sensations, urinary disorders, piles and all kinds of inflammation [8]. Phytochemical analysis has shown the presence of β-phenethylamine, *N*-methyl-β phenethylamine, *S*-(+)-*N* b-methyltryptophan methylester, vasicine, choline, betaine, ephedrine, *ψ*-ephedrine [9] as the active principles of its aerial parts. Also, presence of sigmasterol, sigmastenol, campesterol, 22-dehydrocampesterol, sitosterol, cholesterol, spinasterol, 24-methylenecholesterol, 22-dihydrospinasterol and some *n*-alkanes and *n*-alcohols have been reported [10,11]. In parts of North East India, a decoction prepared from leaves of SR is consumed by rural and urban populace to alleviate symptoms of obesity and diabetes [12]. Recent studies from our laboratory have reported hypolipidemic [13], anti-diabetic [14] and cardioprotective [15] properties of *S. rhomboidea*. Roxb leaf extract (SRLE) in experimental models. SRLE has also been shown to ameliorate high fat diet (HFD) induced steatohepatitis in C57BL/6J mice [16] and prevents *in vitro* low density lipoprotein oxidation and macrophage apoptosis [17]. Acute and chronic toxicity tests have revealed that SRLE is non-toxic up to a dose of 3000 mg/kg body weight in mice [18].

These findings encouraged us to investigate the anti-obesity potential of SRLE and the possible mechanism thereat. Present study evaluates, the effects of SRLE on the expression of genes associated with adipogenesis, lipolysis and lipogenesis in HFD fed C57BL/6J mice. The focus of this study is on the mRNA expression peroxisome proliferator-activated receptor γ2 (PPARγ2), sterol regulatory element-binding factor 1c (SREBP1c), carnitine palmitol transferase-1(CPT-1), fatty acid synthase (FAS) and leptin in the epididymal adipose tissue of a high fat diet fed C57BL/6J mice. The efficacy of SRLE in controlling *in vitro* adipocyte differentiation and leptin release is also assessed.

## 2. Materials and Methods

### 2.1. Plant Material

Leaves of SR were collected from Imphal district India in the month of June and shade dried. The plant was identified by Dr. Hemchand Singh, Taxonomist, Department of Botany, D.M. College of Science Manipur Imphal and a sample (voucher specimen No. 216) was deposited at the herbarium of the Department of Botany.

### 2.2. Preparation of Extract

For preparation of extract, SR leaves were shade dried, manually crushed and grinded in an electric grinder to obtain fine powder. Hundred gm of powdered leaves were boiled in 1000 mL of distilled water at 100 °C for 3 h and filtered using a sterilized muslin cloth. Resulting filtrate was collected in petri plates and concentrated by heating at 100 °C to form a semisolid paste. This paste was kept overnight at 0 °C and a freeze dried extract was obtained. The net yield obtained at the final step of extract preparation was 24% w/w.

### 2.3. Experimental Animals

Male C57BL/6J mice (6–8 weeks of age) were purchased from National Centre for Laboratory Animal Sciences (NCLAS), National Institute of Nutrition (NIN), Hyderabad, India. They were housed and maintained in clean polypropylene cages and fed with either low fat diet or high fat diet and provided with water *ad libitum*. The experimental protocol was carried out according to the guidelines of the Committee for the Purpose of Control and Supervision of Experiments on Animals (CPCSEA), India and approved by the animal ethical committee of Department of Zoology, The M.S University of Baroda, Vadodara (Approval No. 827/ac/04/CPCSEA).

#### 2.3.1. High Fat Diet Induced Obesity in C57BL/6J Mice

A total of 18 mice were randomly allocated into 3 groups of 6 animals each Group I (LEAN) consisted of mice fed with low fat diet (regular fat diet). Group II (Obese; OB) was fed with high fat diet [16]. Group III (OB + SRLE) consisted of mice fed with high fat diet containing 1% SRLE [14,16]. All animals were fed with their respective diets for 20 weeks.

At the end of the experimental period, overnight fasted animals were given mild ether anesthesia and blood was collected by retro orbital sinus puncture in EDTA coated vials. Plasma was obtained by cold centrifugation (4 °C) of the vials for 10 min at 2000× g. Later, animals were sacrificed by cervical dislocation under mild ether anesthesia and various fat pads were excised and, the epididymal fat pad was stored in RNAlater solution (Ambion) until analysis at −80 °C (Cryo Scientific Ltd., India).

#### 2.3.2. Plasma Lipids and Leptin Assay

Plasma triglycerides (TG) and free fatty acids (FFA) were assayed using commercially available kits (Reckon Diagnostics, Ltd., Baroda, India) whereas; plasma leptin was assayed using anti-mouse monoclonal antibody coated 96 microtiter plate as per the instructions of the manufacturer (KRISHGEN Biosystems, Ltd.).

#### 2.3.3. Analysis of Gene Expression by Quantitative RT-PCR (qPCR)

Total RNA was isolated from the epididymal fat pad of control and experimental mice using Tri-reagent (Sigma Aldrich, USA). Quantity and quality of isolated RNA was assessed using nanodrop spectrophotometer (Thermo Scientific, Ltd.) and samples with a ration of A_260_/A_280_ > 1.9 was processed for cDNA synthesis using Omniscript cDNA synthesis kit (Qiagen, USA). A reaction mixture of 20 μL contained, 2 μg total RNA, 10 × RT buffer, dNTP mixture (5mm each), 10 × random hexamer, RNase inhibitor (10 U/μL), Omniscript RT (4 U/rxn) and RNase free water. The cDNA synthesis was carried out at 37 °C for 1 h using a Veriti 96 well thermal cycler (Applied Biosystems, USA). Real-time PCR assays were performed in 96-well plates in ABI 7500 Fast real-time PCR machine (Applied Biosystems, USA). Primer sequences for qPCR analysis were as follow: PPARγ2 (sense) TCACAAGAGCTGACCCAATG, (anti-sense) GCATCCTTCACAAGCATGAA, SREBP1c (sense) GATCAAAGAGGAGCCAGTGC, (anti-sense) TAGATGGTGGCTGCTGAGTG, FAS (sense) GGGTCTATGCCACGATT, (anti-sense) CACAGGGACCGAGTAATG, CPT-1 (sense) CTCAGTGGGAGCGACTCTTCA, (anti-sense) GGCCTCTGTG GTACACGAC AA, LEP (sense) GACACCAAAACCCTCAT, (anti-sense) CAGAGTCTGGTCCATCT, GAPDH (sense) AGGCCGGTGCTGAGTATGT, (anti-sense) GTGGTTCACACCCATCACAA. Syber Green reaction mixture of 20 μL contained 10 μL Quantifast Syber green master mix (Qiagen, USA), 2 μL template, 1 μL of each primer and 6 μL nuclease free water. The following two steps thermal cycling profile was used for qPCR analysis, Step I (cycling step): 95 °C for 10 min, 95 °C for 15 s, 60 °C for 1 min and 95 °C for 15 s for 40 cycles. Step II (Melt Curve step): 60 °C for 15 s, 60 °C 1 min and 95 °C for 30 s.

The fold change in the expression of genes mentioned herein was done by normalizing the values of threshold cycle (CT) of target gene with the CT value of Housekeeping gene (GAPDH) so as to obtain the ΔCT value. These values were further normalized with ΔCT values of CON (ΔCT_OB or OB+SR_ − ΔCT_LEAN_) so as to obtain ΔΔCT values. The fold change in expression was then obtained log 2 − ΔΔCT [19].

#### 2.3.4. Microscopic and Morphometric Examination of Epididymal Fat Pad

Epididymal fat pad was fixed in 4% buffered paraformaldehyde, dehydrated in graded alcohol series and embedded in paraffin wax using automated tissue processor. Five μm sections were cut and stained with hematoxyline and eosin and examined under Leica microscope. Photographs of adipocyte were taken with Canon power shot S70 digital Camera at 400× magnification and adipocyte number per 1000 mm were calculated using image analysis software.

### 2.4. Maintenance of 3T3L1 Cells

3T3-L1 mouse preadipocytes (Obtained from National Centre for Cell Sciences, Pune, India) were maintained in Dulbaco’s modified eagle medium (DMEM) containing 10% fetal bovine serum (FBS) (Himedia Pvt Ltd., Mumbai, India) and 1% antibiotic-antimycotic solution (10×; (Himedia Pvt Ltd., Mumbai, India) and sub cultured every 3rd day using 0.25% trypsin-EDTA solution (Himedia Pvt Ltd., Mumbai, India).

#### 2.4.1. *In Vitro* Cytotoxicity Assay

Pre-confluent pre-adipocytes (5.0 × 10^3^ cells/well) were maintained in 96 well plates (Tarson India Pvt Ltd.) for 72 h in presence of SRLE (10–1000 μg/mL) or vehicle (0.9% NaCl). At the end of incubation period, 10 μL of 3-(4,5-Dimethylthiazol-2-yl)-2,5-diphenyltetrazolium bromide (MTT; 5 mg/mL) was added to wells and the plates were incubated at 37 °C for 4 h. At the end of incubation, culture media was discarded and the wells were washed with phosphate buffer saline (Himedia Pvt Ltd., Mumbai, India). 150 μL of dimethyl sulphoxide was added to all the wells and were incubated for 30 min. Absorbance was read at 540 nm in ELX800 Universal Microplate Reader (Bio-Tek instruments, Inc., Winooski, VT) and % cell viability was calculated.

#### 2.4.2. *In Vitro* Adipocyte Differentiation Protocol

Freshly sub cultured cells were seeded on 12 well cell culture plates at the density of 1.0 × 10^5^ cell/well in DMEM containing 10% FBS and allowed to become confluent. Cells were maintained for 2 days in confluent stage (To arrest cell division). Later (at day 0), culture media was replaced with DMEM containing 0.5 mM 3-isobutyl-1-methylxanthine (Sigma Aldrich, USA), 0.25 μM dexamethosone (Sigma Aldrich, USA), and 10 μg/mL insulin(Sigma Aldrich, USA) and cells were maintained for 4 days. At the end of 4 days, culture media was replaced with maturation media containing complete DMEM with 10 μg/mL insulin and cells were maintained for another 8 days, subsequently media was replaced every 48 hr till the end of the experiment [20].

#### 2.4.3. Qualitative and Quantitative Analysis of Adipocyte Differentiation

3T3-L1 pre-adipocytes were differentiated as describe above in presence of absence of SRLE (10–200 μg/mL). At the end of 12th day, Oil Red O staining for adipocyte lipid accumulation was performed. At the end of incubation, cells were washed twice with PBS and fixed in a 4% buffered paraformaldehyde for 10 min, washed twice with Milli Q water (Millipore India Pvt Ltd.) and then stained using 0.5% Oil Red O (ORO) for 15 min at room temperature. Excess ORO dye was washed with Milli Q water and photographs were taken in Leica DMIL inverted microscope using Canon power shot S70 digital camera [16].

In another set of experiment the stained adipocytes were treated with 100% isopropanol (to extract intracellular Oil Red O stain) and then the absorbance (Optical density; OD) of the extracts was measured at 490 nm. Reagent blank and cell blank assay was also performed simultaneously to minimize non-specific staining. The difference in absorbance between cells with and without ORO dye was calculated. Percentage adipogenesis was calculated as: (OD of treated cells)/(OD of untreated cells) × 100.

#### 2.4.4. Leptin Release and Triglyceride Accumulation Assays

3T3-L1 pre-adipocytes were differentiated as described above in presence of SRLE (10–200 μg/mL) or vehicle (0.9% NaCl) and leptin and TG contents were assayed in supernatant and cells respectively. On day 12, supernatants from each well were collected and leptin content was analyzed using mouse specific leptin ELISA kit (Krishgen, Biosystems) as per the instructions of the manufacturer. After removal of supernatants, cells were washed twice with Phosphate buffer saline (PBS) and solublized in 100 μL of 1% Triton X 100 (in PBS) and, assayed for total TG using commercially available enzymatic kit (Reckon Diagnostics, Baroda, India) using Merck Microlab L300 Semi-autoanalyzer. Results were expressed as % TG [20].

#### 2.4.5. Glycerol Release Assay

3T3-L1 pre-adipocytes were differentiated as described above for 12 days. For glycerol release assay, differentiated adipocytes were incubated with SRLE (10–200 μg/mL) or vehicle (0.9% NaCl) for 48 h. At the end of incubation, supernatant was collected from each well and glycerol content was determined by spectrophotometric assay [21].

#### 2.4.6. G3PDH Activity Assay

3T3-L1 pre-adipocytes were differentiated as described above in presence of SRLE (10–200 μg/mL) or vehicle (0.9% NaCl). On day 12 after removal of supernatants, cells were washed twice with ice-cold PBS on and lysed in Tris-EDTA buffer (25 mM Tris/1 mM EDTA, pH 7.5) and glyceraldehydes-3-phosphate dehydrogenase (G3PDH) activity was determined [22]. Protein content in the cell lysate was determined using bovine serum albumin as a standard [23].

### 2.5. Statistical Analysis

Statistical evaluation of the data was done by one way ANOVA followed by Bonferroni’s multiple comparison test.The results were expressed as mean ± S.E.M using Graph Pad Prism version 3.0 for Windows, Graph Pad Software, San Diego, CA, USA.

## 3. Results

### 3.1. Body Weight Gain, Food Intake and Feed Efficiency

**Figure 1:**
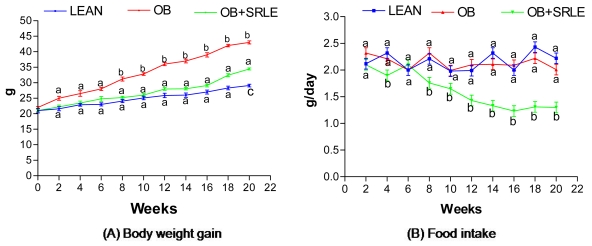
Effect of *S. rhomboidea*. Roxb leaf extract feeding on (**A**) body weight gain and (**B**) food intake. Results are expressed as mean ± S.E.M., *n* = 6. Where, time points not sharing common letter indicate significant differences (*p* < 0.05).

As shown in Figure 1A, HFD fed OB mice recorded significant weight gain after 20 weeks compared to the lean control. However, HFD induced weight gain was significantly controlled by SRLE supplementation of OB mice. There was also time dependent decrement in food intake in SRLE supplemented OB mice (Figure 1B). There was a significant increment in the feed efficiency ratio (body weight gain/food intake) in OB mice compared to LEAN mice (0.071 ± 0.004 *vs*. 0.030 ± 0.002). However, FER was successfully controlled in the OB+SRLE mice compared to OB mice (0.058 ± 0.003 *vs*. 0.071 ± 0.004).

### 3.2. Plasma Lipids and Leptin

OB mice recorded significant increase in plasma TG, FFA and LEP compared to LEAN mice while, SRLE supplementation to HFD fed mice resulted in significant decrement in plasma TG, FFA and LEP compared to OB mice (Table 2).

### 3.3. Visceral Adiposity

**Figure 2:**
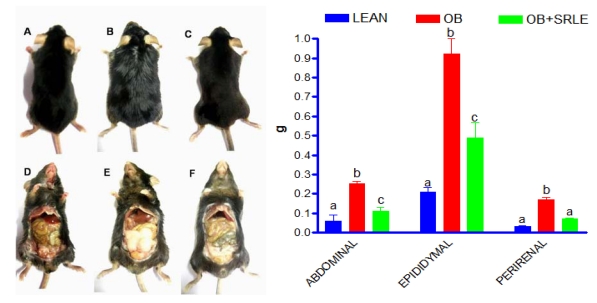
Effect of *S. rhomboidea*. Roxb leaf extract feeding on morphological and anatomic evaluation of visceral adiposity in Lean (**A** and **D**), OB (**B** and **E**) and OB + SRLE (**C** and **F**) groups and abdominal, epididymal and perirenal fat pad weights. Results are expressed as mean ± S.E.M., *n* = 6. Where, timepoints not sharing common letter indicate significant differences (*p* < 0.05).

Visceral adiposity was evident in the form of increase in various fat pad weights (Perirenal, abdominal and epididymal) in OB mice after 20 weeks of HFD feeding (Figure 2). OB mice supplemented with SRLE extract showed significant attenuation in HFD induced visceral adiposity and increment in fat pad weights (Figure 2).

### 3.4. Microscopic and Morphometric Evaluation of Epididymal Fat Pad

Microscopic evaluation of epididymal fat pad of OB mice recorded adipocyte hypertrophy that was marked by significant reduction in the adipocyte number (120.0 ± 12.0 *vs*. 270.0 ± 19.00) per 1000 mm area compared to LEAN. However, OB+SR mice recorded significantly higher number of adipocytes per (170.0 ± 15.0 *vs*. 120.0 ± 12.0) 1000 mm area compared to OB.

### 3.5. Quantitative RT PCR Analysis

**Figure 3:**
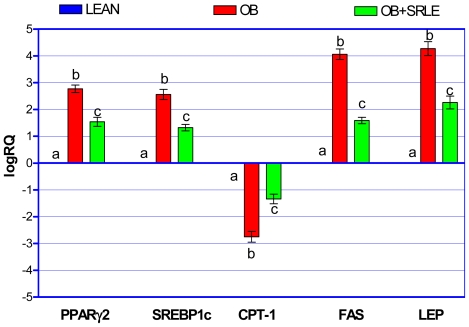
Effect of S.rhomboidea.Roxb leaf extract feeding on quantitative RT-PCR analysis of PPARγ2, SREBP1c, CPT-1 FAS and LEP mRNA expression. Results are expressed as mean ± S.E.M., *n* = 6. Where, timepoints not sharing common letter indicate significant differences (*p* < 0.05).

HFD fed OB mice recorded significantly up-regulated mRNA expression of PPARγ2, SREBP1c, FAS and LEP, while CPT-1 expression was significantly down-regulated in epididymal fat pad compared to lean mice. However, these set of changes were significantly prevented in SRLE supplemented OB mice (Figure 3).

### 3.6. Cytotoxicity Assay

**Figure 4:**
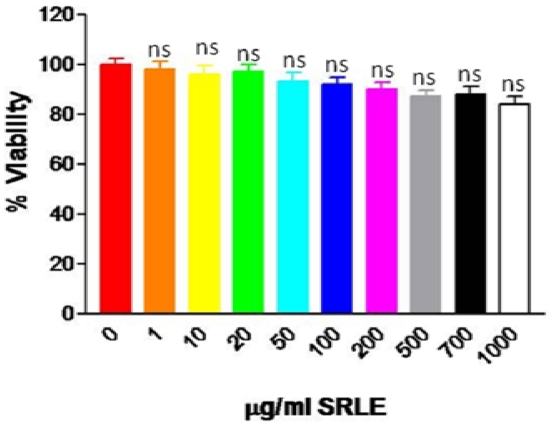
Effect of *S.rhomboidea*.Roxb leaf extract on cell viability. Results are expressed as mean ± S.E.M., *n* = 3. Where, * *P* < 0.05, ** *P* < 0.01 and *P* < 0.001 and ^ns^ non significant compared to 0 μg/mL SRLE.

Cytotoxicity analysis of SRLE in pre-adipocyte cells registered non significant alteration in the cell viability in the dose range of 10–1000 μg/mL (Figure 4).

### 3.7. Qualitative and Quantitative Analysis of Adipocyte Differentiation

**Figure 5:**
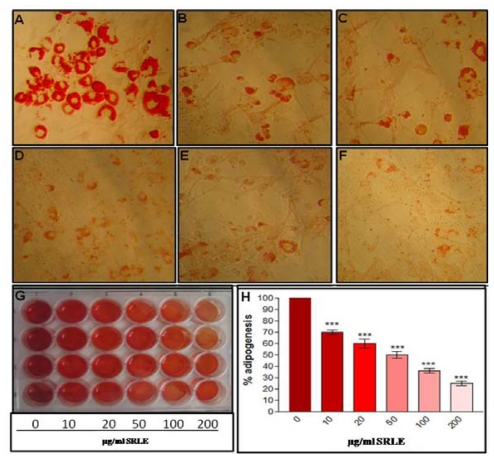
Photomicrograph of Oil Red O stained differentiating 3T3L1 cells (**A**) untreated, (**B**) treated with 10 μg/mL SRLE, (**C**) treated with 20 μg/mL SRLE, (**D**) treated with 50 μg/mL SRLE, (**E**) treated with 100 μg/mL SRLE and (**F**) treated with 200 μg/mL SRLE and qualitative (whole well image of Oil Red O stained adipocytes) and quantitative (% adipogenesis) evaluation of adipocyte differentiation (**G** and **H**). Results are expressed as mean ± S.E.M., *n* = 3. Where, * *P* < 0.05, ** *P* < 0.01 and *P* < 0.001 and ^ns^ non significant compared to 0 μg/mL SRLE.

ORO staining of differentiated adipocytes at the end of 12 days revealed significant cytoplasmic lipid accumulation in the untreated differentiated adipocytes (Figure 5A) whereas, SRLE extract supplementation to differentiating 3T3L1 pre-adipocytes significantly reduced adipocyte differentiation, characterized by lesser cytoplasmic lipid accumulation (Figure 5B–F). Quantitative analysis of ORO staining recorded 30% to 75% inhibition in adipogenesis in the SRLE supplemented 3T3L1 cells.

### 3.8. Trigyceride Accumulation and Leptin Release in Differentiated Adipocytes

**Figure 6:**
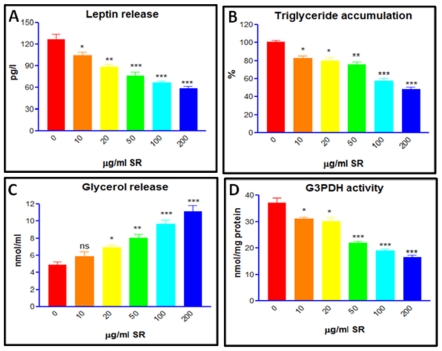
Effect of *S. rhomboidea*. Roxb leaf extract on *in vitro* (**A**) leptin release, (**B**) triglyceride accumulation, (**C**) glycerol release, and (**D**) G3PDH activity during 3T3L1 pre-adipocyte differentiation were evaluated as described in materials and methods. Results are expressed as mean ± S.E.M., *n* = 3. Where, * *P* < 0.05, ** *P* < 0.01 and *P* < 0.001 and ^ns^ non significant compared to 0 μg/mL SRLE.

Figure 6A,B shows LEP release and TG accumulation in the untreated and SRLE supplemented differentiated adipocytes at the end of 12 days. Whereas, untreated adipocytes showed higher levels of LEP release and TG accumulation compared to pre-adipocytes (Figure 6A,B). SRLE supplementation to differentiating adipocytes recorded significant decrement in the LEP release and TG accumulation compared to differentiated untreated adipocytes (Figure 6A,B).

### 3.9. Glycerol Release and G3PDH Activity Assay

SRLE supplementation to differentiating pre-adipocytes resulted in higher indices of glycerol release and lowered cellular G3PDH activity compared to untreated differentiated adipocytes (Figure 6C,D).

## 4. Discussion

The onset and progression of HFD induced experimental obesity in C57BL/6J mice has a physiological resemblance to high calorie diet induced obesity in humans [24]. The high fat diet induced obesity in C57BL/6J mice characterized by selective deposition of fat in the mesentery, is comparable to visceral adiposity in humans [25,26]. Hence, this experimental model is widely used to investigate anti-obesity potential of herbal formulations [27,28]. Significant prevention of body weight gain and reduction in food intake caused due to SRLE supplementation of HFD fed mice recorded in the present study are in agreement with our previous observations [13]. SRLE induced decrement in food intake could be attributed to presence of ephedrine and pseudo-ephedrine in various species of *Sida* [29], including *Sida rhomboidea.* Roxb [9,30]. Hence, it is assumable that, ephedrine induced appetite suppression is the key aspect that inhibits body weight gain in SRLE supplemented OB mice. Previous studies have reported that, ephedrine promotes the release of noradrenaline and inhibits the uptake of noradrenalin that eventually; results in decreased food intake and satiety [31,32]. However, mechanism responsible for SRLE induced decreased food intake needs further investigation. Ephedrine content of SRLE is <0.01% and, in the present study, it amounts to 50 μg/kg body weight, a dose that is almost 400 times lesser than the upper limit toxic dose (24 mg/kg body weight/day) set by USFDA [33]. Further, the safety evaluation of SRLE reported from our laboratory had shown that dose of 3000 mg/kg body weight is non-toxic in *Swiss* albino mice [18].

Adipose tissue represents a dynamic endocrine organ that not only maintains energy balance but also controls lipid homeostasis mainly by two reciprocal processes; lipogenesis and lipolysis [34]. Moreover, adipogenesis is a complex process in which both adipocyte growth and differentiation contribute to overall adipose mass [35]. Therefore, adipocyte differentiation is crucial in the maintenance of adipose tissue integrity. The net balance between the three processes determines the onset of visceral obesity. In the present study, OB mice recorded significantly increased mass of various fat pads. These results are further substantiated by concomitant decrement in adipocyte number, due to augmented TG deposition and adipocyte hypertrophy. The observed changes in epididymal fat pad of OB mice are in agreement with the recorded elevation in plasma leptin titre and the up-regulated expression of leptin mRNA in adipose tissue. However, SRLE supplementation of HFD fed mice show higher number of non-atrophied adipocytes, decrement in fat pad weights and plasma TG levels coupled with lower plasma leptin titer and down-regulation of epididymal leptin mRNA expression compared to OB mice. Since, plasma leptin and its mRNA expression are synonymous with obesity [36] and TG induced adipocyte hypertrophy [37], the results recorded herein are indicative of the mitigative nature of SRLE against HFD induced visceral adiposity, essentially by altered leptin gene expression in adipocytes.

The sequence of events beginning with adipocyte differentiation and ending with obesity is marked by up-regulation of prominent adipogenic genes [38]. PPARγ2, a nuclear receptor is a transcription factor that plays a crucial role in stimulating differentiation of pre-adipocytes and progenitor cells into adipocytes [39]. Recent studies have shown that some of the lipid lowering plant extracts acts as PPARγ2 antagonists thus preventing fat accumulation and adipocyte differentiation [40,41]. SREBP1c is another adipogenic transcription factor that controls the production of endogenous ligands for PPARγ [42] thus regulating the downstream cascade of lipid homeostasis and adipogenesis [43]. SREBP1c mediated lipogenesis in hepatic and adipose tissue is differential [44]. PPARγ2 has been reported to mediate lipogenesis in adipose tissue either directly or via modulation of SREBP1c [45,46]. In the present study, OB mice recorded significant up-regulation of PPARγ2 and SREBP-1c expression in the epididymal adipose tissue. However, SRLE supplementation not only prevented the up-regulated expression of these genes but also attenuated adipogenesis.

FAS gene expression is governed by PPARγ2 and is involved in the facilitated conversion of FFA into TG in adipocytes thus contributing to adipocyte hypertrophy and visceral adiposity in HFD fed OB mice [47]. This physiological condition is parallel by with elevated plasma levels of TG and FFA. However, the recorded relatively lowered plasma TG and FFA levels in SRLE supplemented HFD fed mice is attributable to the reduced food intake of these animals. Further, the down regulation of FAS in adipocytes can again be correlated with lower expression of PPARγ2 in SRLE supplemented OB mice and the resultant anti-adipocyte hypertrophy. In the present study, HFD fed OB mice recorded significant up-regulation of FAS expression along with down-regulation of CPT-1 expression thus shifting the overall balance towards lipid anabolism. However, up-regulation of CPT-1 expression observed in SRLE supplemented OB mice along with down-regulation of PPARγ2 and FAS provides ample evidence for the anti-lipogenic potential of SRLE. Also, previous studies have documented PPARγ2 induced down regulation of ACC along with up-regulation of CPT-1 expression that eventually controls visceral adiposity [48]. Hence, inhibitory effect of SRLE on expression of ACC can not be ruled out and requires further scrutiny. It is inferable from the results observed herein that, the anti-obesity property of SRLE is a two pronged process that acts via reduction of dietary intake and down regulation of PPARγ2.

Anti-obesity potential of herbal extracts is also assessed *in vitro* using 3T3L1 pre-adipocytes as; these cells accumulate TG and release LEP when they differentiate into adipocytes [49,50]. In the present study, experimentally induced adipocyte differentiation was characterized by plumping of adipocytes due to accumulation of red colored lipid droplets and higher percentage of adipocyte differentiation. Also, TG accumulation and G3PDH activity level were all elevated in untreated adipocytes. The fully differentiated state of adipocytes was confirmed by the augmented released of leptin from these cells. However, in the presence of SRLE, differentiating adipocytes results in an overall decrement in the qualitative and quantitative indices. Qualitatively, the inhibitory effect of SRLE on adipocyte differentiation was marked by admixture of undifferentiated and differentiated cells and minimal lipid accumulation. These observations were further substantiated by the dose dependent reduction in TG content, G3PDH activity and LEP titer. Further, treatment of SRLE to differentiated adipocytes resulted in dose dependent increment in the glycerol release. These findings provide unequivocal evidence for the potential of SRLE in preventing adipocyte differentiation, lipid accumulation and LEP release and promoting lipolysis.

## 5. Conclusion

It can be concluded from the present study that the mechanism governing the anti-obesity potential of SRLE is a two pronged process that involves: (i) attenuation of food intake; and (ii) down regulation of PPARγ2 and related lipogenic genes that control visceral adiposity. These contentions are substantiated by the demonstration of SRLE induced prevention of *in vitro* adipocyte differentiation and leptin release. However, detailed scrutiny of its active phyto-ingredients along with clinical investigations is warranted to develop a herbal anti-obesity formulation.

## Figures and Tables

**Table 1:** Composition of experimental diets.

Ingredients	Low Fat Diet (g/kg)	High Fat Diet (g/kg)

Casein	200	200
l-Cystine	3	3
Corn Starch	315	0.0
Maltodextrin	35	125
Sucrose	100	68.8
Cellulose	50	50
Soybean Oil	25	25
Lard	20	245
Mineral Mix 1	10	10
Di Calcium Phosphate	13	13
Calcium Carbonate	5.5	5.5
Potassium Citrate	16.5	16.5
Vitamin Mix 2	10	10
Choline chloride	2	2
Regular chow	195	216.25

**SRLE**	00	10

***Energy Content***	16.25 kJ/g	25.72 kJ/g
Protein, % of energy	20	20
Carbohydrate, % of energy	64	35
Fat, % of energy	6	45

1 Mineral mix adds the following components (per g mineral mix): sodium chloride, 259 mg; magnesium oxide, 41.9 mg; magnesium sulfate, 257.6 mg; chromium K sulfate, 1.925 mg; cupric carbonate, 1.05 mg; sodium fluoride, 0.2 mg; potassium iodate, 0.035 mg; ferric citrate, 21 mg; manganous carbonate, 12.25 mg; ammonium molybdate, 0.3 mg; sodium selenite, 0.035 mg; zinc carbonate, 5.6 mg;

2 Vitamin mix adds the following components (per g vitamin mix): retinyl acetate, 0.8 mg; cholecalciferol, 1.0 mg; dl-a-tocopheryl acetate, 10.0 mg; menadione sodium bisulfite, 0.05 mg; biotin, 0.02 mg; cyanocobalamin, 1 mg; folic acid, 0.2 mg; nicotinic acid 3 mg; calcium pantothenate, 1.6 mg; pyridoxine-HCl, 0.7 mg; riboflavin, 0.6 mg; thiamin HCl, 0.6 mg.

**Table 2:** Effect of *S. rhomboidea*. Roxb leaf extract feeding on plasma triglycerides, free fatty acids and leptin titer.

	LEAN	OB	OB+SRLE
**Plasma**			
Triglycerides (mmol/L)	0.53 ± 0.02^a^	2.16 ± 0.05^b^	0.61 ± 0.04^a^
Free fatty acids (mmol/L)	1.80 ± 0.07^a^	4.68 ± 0.09^b^	2.33 ± 0.08^a^
Leptin (ng/L)	12.00 ± 1.98^a^	46.24 ± 2.13^b^	25.01 ± 1.89^c^

Results are expressed as mean ± S.E.M., *n* = 6. Where, timepoints not sharing common letter indicate significant differences (*p* < 0.05).

## References

[1] World Health Organization (2010). World Health Organization Fact Sheet for Worldwide Prevalence of Obesity.

[2] Haffner SM (2006). Abdominal obesity, insulin resistance, and cardiovascular risk in pre-diabetes and type 2 diabetes. Eur. Heart J.

[3] Schuster DP (2010). Obesity and the development of type 2 diabetes: The effects of fatty tissue inflammation. Diabetes Metab. Syndr. Obes.

[4] Barsh GS, Farooqi IS, O’Rahilly S (2000). Genetics of body-weight regulation. Nature.

[5] Bråkenhielm E, Cao R, Gao B, Angelin B, Cannon B, Parini P, Cao Y (2004). Angiogenesis inhibitor, TNP-470, prevents diet-induced and genetic obesity in mice. Circ. Res.

[6] Yun JW (2010). Possible anti-obesity therapeutics from nature–A review. Phytochemistry.

[7] Kang S, Kim MH, Shin HS, Kim HM, Hong YS, Park JG, Ko HC, Lee NH, Chung WS, Kim SJ (2010). A water-soluble extract of *Petalonia binghamiae* inhibits the expression of adipogenic regulators in 3T3-L1 pre-adipocytes and reduces adiposity and weight gain in rats fed a high-fat diet. J. Nutr. Biochem.

[8] Ramachandra Rao R, Sudarshan SK (2005). Encyclopaedia of Indian Medicine.

[9] Prakash A, Verma RK, Ghosal S (1981). Alkaloidal constituents of *Sida acuta*, *S. rhombifolia* and *S. spinosa*. Plant. Med.

[10] Goyal MM, Rani KK (1988). Effect of natural products isolated from three species of *Sida* on some gram-positive and gran-negetive bacteria. J. Ind. Chem. Soc.

[11] Goyal MM, Rani KK (1989). Neutral constituents of the aerial parts of *Sida rhombifolia* var. *rhomboidea*. Fitoterapia.

[12] Thounaojam MC, Jadeja RN, Ansarullah, Devkar RV, Ramachandran AV (2009). Potential of *Sida rhomboidea*. Roxb leaf extract in controlling hypertriglyceridemia in experimental models. Pharmacogn. Res.

[13] Thounaojam M, Jadeja R, Ansarullah, Devkar R, Ramachandran AV (2009). Dysregulation of lipid and cholesterol metabolism in high fat diet fed hyperlipidemic rats: Protective effect of *Sida rhomboidea*. Roxb leaf extract. J. Health Sci.

[14] Thounaojam MC, Jadeja RN, Ansarullah, Devkar RV, Ramachandran AV (2010). Prevention of high fat diet induced insulin resistance in C57BL/6J mice by *Sida rhomboidea*. Roxb extract. J. Health Sci.

[15] Thounaojam MC, Jadeja RN, Ansarullah, Karn SS, Shah JD, Patel DK, Salunke SP, Padate GS, Devkar RV, Rmachandran AV (2011). Cardioprotective effect of *Sida rhomboidea*. Roxb extract against isoproterenol induced myocardial necrosis in rats. Exp. Toxicol. Pathol.

[16] Thounaojam MC, Jadeja RN, Dandekar DS, Devkar RV, Ramachandran AV (2010). *Sida rhomboidea. *Roxb extract alleviates pathophysiological changes in experimental *in vivo* and *in vitro* models of high fat diet/fatty acid induced non-alcoholic steatohepatitis. Exp Toxicol Pathol.

[17] Thounaojam MC, Jadeja RN, Devkar RV, Ramachandran AV (2011). *In Vitro* evidence for the protective role of *Sida rhomboidea*. Roxb extract against LDL oxidation and oxidized LDL-induced apoptosis in human monocyte-derived macrophages. Cardiovasc. Toxicol.

[18] Thounaojam MC, Jadeja RN, Patel DK, Devkar RV, Ramachandran AV (2010). Acute and sub chronic oral toxicity of *Sida rhomboidea.*Roxb leaf extract. J Complement Integr Med.

[19] Kumar TP, Antony S, Gireesh G, George N, Paulose CS (2010). Curcumin modulates dopaminergic receptor, CREB and phospholipase c gene expression in the cerebral cortex and cerebellum of streptozotocin induced diabetic rats. J. Biomed. Sci.

[20] Jadeja RN, Thounaojam MC, Ramani UV, Devkar RV, Ramachandran AV (2011). Anti-obesity potential of *Clerodendron glandulosum*. Coleb leaf aqueous extract. J. Ethnopharmacol.

[21] Sturgeon RJ, Deamer RL, Harbison HA (1979). Improved spectrophotometric determination of glycerol and its comparison with an enzymatic method. J. Pharm. Sci.

[22] Wise LS, Green H (1979). Participation of one isozyme of cytosolic glycerophosphate dehydrogenase in the adipose conversion of 3T3 cells. J. Biol. Chem.

[23] Lowry OH, Rosebrough NJ, Farr AL, Randall RJ (1951). Protein measurement with the folin phenol reagent. J. Biol. Chem.

[24] West KM, Kalbfleisch JM (1971). Influence of nutritional factors on prevalence of diabetes. Diabetes.

[25] Rebuffe-Scrive M, Surwit R, Feinglos M, Kuhn C, Rodin J (1993). Regional fat distribution and metabolism in a new mouse model (C57BL/6J) of non-insulin-dependent diabetes mellitus. Metabolism.

[26] Surwit RS, Feinglos MN, Rodin J, Sutherland A, Petro AE, Opara EC, Kuhn CM, Rebuffé-Scrive M (1995). Differential effects of fat and sucrose on the development of obesity and diabetes in C57BL/6J and A/J mice. Metabolism.

[27] Kim MJ, Kim HK (2009). Perilla leaf extract ameliorates obesity and dyslipidemia induced by high-fat diet. Phytother. Res.

[28] Kim NH, Choi SK, Kim SJ, Moon PD, Lim HS, Choi IY, Na HJ, An HJ, Myung NY, Jeong HJ, Um JY, Hong SH, Kim HM (2008). Green tea seed oil reduces weight gain in C57BL/6J mice and influences adipocyte differentiation by suppressing peroxisome proliferator-activated receptor-gamma. Pflugers Arch.

[29] Singh AP (2006). Bala (*Sida cordifolia* L.)—Is it safe herbal drug?. Ethnobot. Leafl.

[30] Khatoon S, Srivastava M, Rawat AKS, Mehrotra S (2005). HPTLC method for chemical standardization of *Sida* species and estimation of the alkaloid ephedrine. J. Planar Chromatogr.

[31] Astrup A, Breum L, Toubro S (1995). Pharmacological and clinical studies of ephedrine and other thermogenic agonists. Obes. Res.

[32] Carek PJ, Dickerson LM (1999). Current concepts in the pharmacological management of obesity. Drugs.

[33] Cristin M (1997). Ephedra falls under FDA jurisdiction—natural stimulant.

[34] Marques BG, Hausman DB, Martin RJ (1998). Association of fat cell size and paracrine growth factors in development of hyperplastic obesity. Am. J. Physiol.

[35] Garaulet M, Hernandez-Morante JJ, Lujan J, Tebar FJ, Zamora S (2006). Relationship between fat cell size and number and fatty acid composition in adipose tissue from different fat depots in overweight/obese humans. Int. J. Obes.

[36] Maffei M, Halaas J, Ravussin E, Pratley RE, Lee GH, Zhang Y, Fei H, Kim S, Lallone R, Ranganathan S (1995). Leptin levels in human and rodent: measurement of plasma leptin and ob RNA in obese and weight-reduced subjects. Nat. Med.

[37] Staiger H, Tschritter O, Machann J, Thamer C, Fritsche A, Maerker E, Schick F, Haring HU, Stumvoll M (2003). Relationship of serum adiponectin and leptin concentrations with body fat distribution in humans. Obes. Res.

[38] Nerurkar PV, Lee YK, Nerurkar VR (2010). *Momordica charantia* (bitter melon) inhibits primary human adipocyte differentiation by modulating adipogenic genes. BMC Complement Altern Med.

[39] Rousseau V, Becker DJ, Ongemba LN, Rahier J, Henquin JC, Brichard SM (1997). Developmental and nutritional changes of ob and PPAR gamma 2 gene expression in rat white adipose tissue. Biochem. J.

[40] Christensen KB, Minet A, Svenstrup H, Grevsen K, Zhang H, Schrader E, Rimbach G, Wein S, Wolffram S, Kristiansen K, Christensen LP (2009). Identification of plant extracts with potential anti-diabetic properties: effect on human peroxisome proliferator activated receptor (PPAR), adipocyte differentiation and insulin-stimulated glucose uptake. Phytother. Res.

[41] Freise C, Erben U, Neuman U, Kim K, Zeitz M, Somasundaram R, Ruehl M (2010). An active extract of *Lindera obtusiloba* inhibits adipogenesis via sustained Wnt signaling and exerts anti-inflammatory effects in the 3T3-L1 pre-adipocytes. J. Nutr. Biochem.

[42] Brown MS, Goldstein JL (1997). The SREBP pathway: regulation of cholesterol metabolism by proteolysis of a membrane-bound transcription factor. Cell.

[43] Hsuy SC, Huang CJ (2006). Reduced fat mass in rats fed a high oleic acid–rich safflower oil diet is associated with changes in expression of hepatic PPARα and adipose SREBP-1c–regulated genes. J. Nutr.

[44] Kersten S (2001). Mechanism of nutritional and hormonal regulation of lipogenesis. EMBO Rep.

[45] Kim JB, Wright HM, Wright M, Spiegelman BM (1998). ADD1/SREBP1 activates PPAR gamma through the production of endogenous ligand. Proc. Natl. Acad. Sci. USA.

[46] Farmer SR (2005). Regulation of PPARγ activity during adipogenesis. Int. J. Obes.

[47] Schachtrup CET, Bleck B, Sandqvist A, Spener F (2004). Functional analysis of peroxisome-proliferator-responsive element motifs in genes of fatty acid-binding proteins. Biochem. J.

[48] Seo JB, Choe SS, Jeong HW, Park SW, Shin HJ, Choi SM, Park JY, Choi EW, Kim JB, Seen DS (2011). Anti-obesity effects of *Lysimachia foenum-graecum* characterized by decreased adipogenesis and regulated lipid metabolism. Exp. Mol. Med.

[49] Hong JH, Hwang EY, Kim HJ, Jeong YJ, Lee IS (2009). *Artemisia capillaris* inhibits lipid accumulation in 3T3-L1 adipocytes and obesity in C57BL/6J mice fed a high fat diet. J. Med. Food.

[50] Kubota H, Kojima-Yuasa A, Morii R, Huang X, Norikura T, Rho SN, Matsui-Yuasa I (2009). Anti-obesity effect of *Blumea balsamifera* extract in 3T3-L1 pre-adipocytes and adipocytes. Am. J. Chin. Med.

